# Experimental Investigation of a 300 kW Organic Rankine Cycle Unit with Radial Turbine for Low-Grade Waste Heat Recovery

**DOI:** 10.3390/e21060619

**Published:** 2019-06-23

**Authors:** Ruijie Wang, Guohua Kuang, Lei Zhu, Shucheng Wang, Jingquan Zhao

**Affiliations:** 1 School of Aeronautic Science and Engineering, Beihang University, Beijing 100191, China; 2 Beijing Huahang Shengshi Energy Technology Co., Ltd., Beijing 100191, China; 3 Key Laboratory of Condition Monitoring and Control for Power Plant Equipment, Ministry of Education, North China Electric Power University, Beijing 102206, China

**Keywords:** waste heat recovery, organic Rankine cycle (ORC), heat source temperature and volume flow rate, single-stage radial turbine, electric power output, isentropic efficiency

## Abstract

The performance of a 300 kW organic Rankine cycle (ORC) prototype was experimentally investigated for low-grade waste heat recovery in industry. The prototype employed a specially developed single-stage radial turbine that was integrated with a semi-hermetic three-phase asynchronous generator. R245fa was selected as the working fluid and hot water was adopted to imitate the low-grade waste heat source. Under approximately constant cooling source operating conditions, variations of the ORC performance with diverse operating parameters of the heat source (including temperature and volume flow rate) were evaluated. Results revealed that the gross generating efficiency and electric power output could be improved by using a higher heat source temperature and volume flow rate. In the present experimental research, the maximum electric power output of 301 kW was achieved when the heat source temperature was 121 °C. The corresponding turbine isentropic efficiency and gross generating efficiency were up to 88.6% and 9.4%, respectively. Furthermore, the gross generating efficiency accounted for 40% of the ideal Carnot efficiency. The maximum electric power output yielded the optimum gross generating efficiency.

## 1. Introduction

Global primary energy consumption showed strong growth in 2017, the fastest growth period since 2013, according to a statistical review of world energy by BP p.l.c. [1]. Correspondingly, the carbon emissions caused by energy consumption also increased after showing no or little increment from 2014 to 2016. If production were to continue at this rate, the time that the remaining reserves would last for oil, gas, and coal would be 50.2, 52.6, and 134 years, respectively [1]. Apparently, increasing energy consumption not only results in fossil fuel shortage, but presents a series of severe environmental issues, such as global warming, ozone depletion, and air pollution [2]. With the dual pressures of the energy crisis and environmental issues, it is extremely urgent and significant to explore renewable energy and improve the utilization efficiency of current energy.

Relevant statistics reveal that 50% or more of the total heat generated in industry is low-grade waste heat, most of which is dissipated due to the scarcity of efficient recovery solutions [3]. Afterward, low-grade waste heat sources are regarded as alternative energy sources [2]. Increasing attention has been paid to waste heat recovery, and diverse solutions have been put forward, including the organic Rankine cycle (ORC), supercritical Rankine cycle, Kalina cycle, Goswami cycle, and trilateral flash cycle [4]. Compared with other cycles, the ORC has the merits of high reliability, simple structure, convenient maintenance [5], and environmental friendliness. Therefore, the ORC is progressively accepted as the premier technology for low-grade waste heat recovery [6] and its technology can be applied in heat-to-power conversion from miscellaneous heat sources covering industrial waste heat, geothermal energy, solar thermal energy, biomass energy, and ocean thermal energy [7]. It also shows great potential for relaxing fossil fuel consumption and mitigating environmental issues. Table 1 lists the ORC classification according to the heat source temperature and power capacity range [8].

Unlike the traditional steam Rankine cycle, ORC employs organic substances as working fluid. Considerable research has been published on organic working fluid screening, which has a remarkable impact on the performance of ORC. According to the slope of the vapor saturation curve in the T–s diagram, working fluids are categorized into three groups: wet fluids with negative slope, isentropic fluids with nearly infinite slope, and dry fluids with positive slope [3]. Moreover, it is suggested that desirable working fluids generally have the characteristics of better thermodynamic properties, low toxicity, controllable flammability, good material compatibility and fluid stability [9], and especially lower global warming potential (GWP) and zero ozone depletion potential (ODP). However, none of the working fluids can satisfy all these requirements simultaneously. Therefore, working fluid selection should be incorporated into the specific design and analysis of the ORC. In general, isentropic and dry working fluids are more appropriate for the ORC system to eliminate the possibility of liquid droplets impinging on turbine blades during expansion, and there is no need for a superheated device [10]. Moreover, in order to overcome the disadvantage of temperature mismatching between evaporator and condenser and reduce the irreversibility of the ORC system, some studies chose mixtures as the working fluid [11,12] so that heat transfer in the evaporator could occur under conditions of constant pressure and variable temperature. R245fa, R123, and R134a are the most preferred working fluids in previous research on the ORC [13].

As a device that converts heat to power, the expander, which is crucial in an ORC system, has undergone intensive investigations [14,15,16]. Expanders applied in ORC systems can be classified into two categories: volume-based expanders, comprising scroll, screw, piston, and rotary vane expanders; and velocity-based expanders, including radial and axial flow turbines [15]. Generally, expander selection strongly depends on ORC operating conditions, power output capacity, and working fluid category [17]. Most of the available research focused on utilization of the scroll expander, radial inflow turbine, and screw expander. The power output capacity of a scroll expander ranges from 0.35 to 7.5 kW, followed by a screw expander, which has a power output capacity ranging from 7 to 50 kW, while a turbine can operate over a wide range from the kilowatt to megawatt scale [18].

Table 2 illustrates most of the ORC experimental results, among which the heat source temperature was below 150 °C. It can be seen that various kinds of heat sources were adopted, including water, oil, steam, gas, and electric heaters. However, most of these demonstrations were limited to micro- to mini-scale power output capacity. Moreover, the expander isentropic efficiency was generally lower than 85%. The present paper reports the performance of a 300 kW ORC prototype whose power capacity is much larger than those listed in Table 2. The study aims to further explore the power generating potential of the ORC unit for utilizing low-grade waste heat sources.

In the present study, preliminary experimental research was carried out on an R245fa-based ORC unit with a nominal power capacity of 300 kW, which was designed to recover the waste heat of the cooling water in industry. Considering fluctuations in the parameters of waste heat sources in industry production, variations of the ORC performance with heat source temperature and volume flow rate were investigated. Hot water produced by a boiler was imitated as the low-grade waste heat source. A single-stage radial turbine was employed to convert heat to power, which was inspired by the aviation turbine used in aircraft environmental control systems and coupled with a three-phase asynchronous generator inside a hermetic casing instead of a fan.

## 2. Experimental Apparatus and Equipment

**Figure 1:**
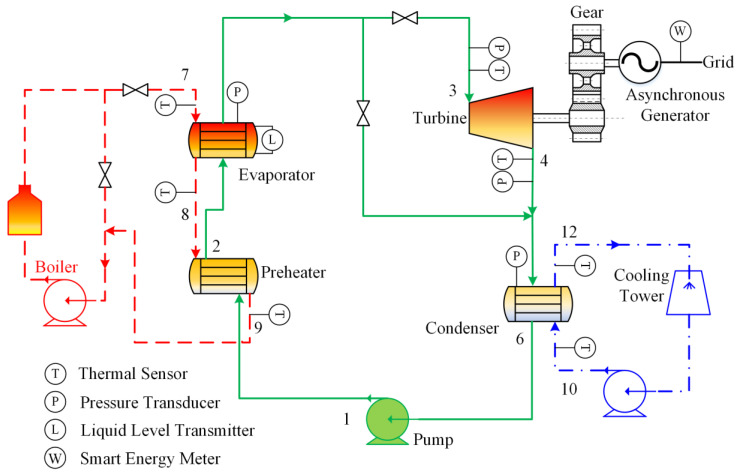
Schematic diagram of the ORC experimental system.

**Figure 2:**
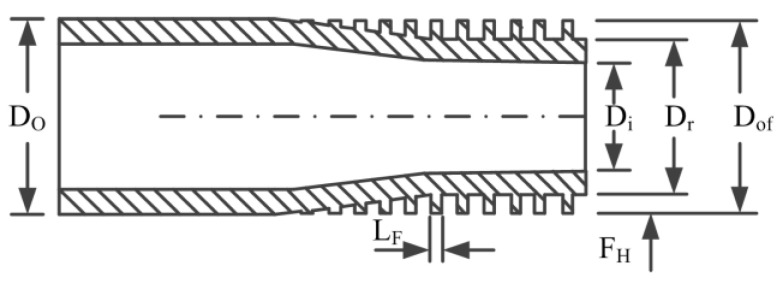
Schematic chart of the low-finned tube in preheater and evaporator.

**Figure 3:**
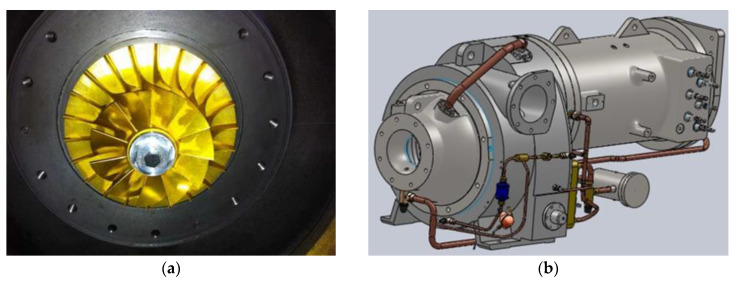

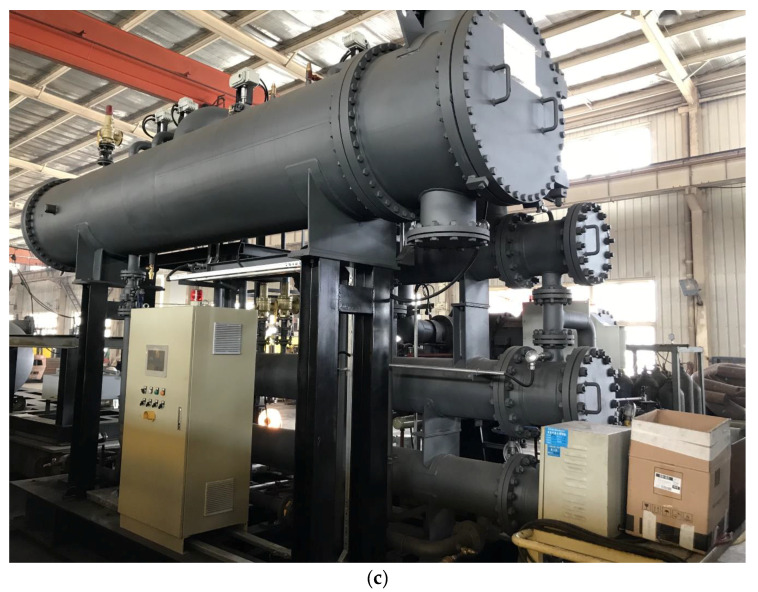
Photographs of (**a**) radial turbine; (**b**) integrated unit of turbine and generator; (**c**) ORC prototype.

Experimental investigations were conducted on a 300 kW ORC unit located in Hefei, China. The experimental apparatus consists of a preheater and a condenser of shell-and-tube type, a flooded evaporator, a radial turbine integrated with a semi-hermetic three-phase asynchronous generator, and a centrifugal pump. Figure 1 depicts the schematic diagram of the ORC prototype. There are three main loops in the thermodynamic process: heating source loop, ORC loop, and cooling source loop. The schematic chart of low-finned tube employed in preheater and evaporator is described in Figure 2 while Figure 3 shows photographs of the ORC experimental apparatus. 

As mentioned in Table 2, R245fa and R123 are commonly utilized in the experimental investigations of ORC for low-grade waste heat recovery. However, R123 was excluded due to its non-zero ODP, as listed in Table 3, whereas R245fa was selected as the working fluid in the present experimental investigation, due to its excellent thermo–physical properties and environmentally-friendly characteristics.

### 2.1. Heating Source Loop

Pressurized hot water without phase transition produced by a boiler was used to emulate the low-grade heat source, and rejected heat to R245fa while passing through the evaporator and preheater in sequence. The heat source temperature was controlled in the range of 101 to 121 °C. An adaptive control technique was adopted to regulate the heat source volume flow rate on the basis of parameters such as generating capacity and heat source temperature.

### 2.2. ORC Loop

Three heat exchangers assembled in the ORC prototype were shell-and-tube exchangers. R245fa flowed on the shell side of the preheater, evaporator, and condenser. A magnetic float liquid level sensor was installed outside of the evaporator and transmitted corresponding electrical signals to the control cabinet. 

Inspired by aviation turbine technology and taking the thermodynamic properties of R245fa into account, numerical simulation was performed on the three-dimensional turbine model in CFD (computational fluid dynamics) software. Based on the simulation results, optimization was conducted on the design of impeller and volute. Then the single-stage radial turbine was specially designed and integrated with a three-phase asynchronous generator, as shown in Figure 3b. Moreover, the higher isentropic efficiency was verified by a series of tests. The turbine shaft power was transmitted to the generator via a gear box with a rotating ratio of 6:1. The bypass valve was in open position to ensure smooth working condition of the turbine in the start-up stage and prevent the turbine from overloading in the operation stage. 

A vertical multi-stage centrifugal pump was employed to keep R245fa circulating in the ORC loop. The maximum working pressure and volume flow rate of the pump were 2.5 MPa and 30 m^3^/h, respectively. A frequency converter was used to adjust the pump frequency; as a consequence, the volume flow rate of R245fa was regulated to make sure that the liquid level in the evaporator was within the permitted range.

### 2.3. Cooling Source Loop

After the cooling water took the heat away from the R245fa in the condenser, it went through a spray-cooling process in a cooling tower, which dissipated the heat to the ambient air. Then the cooling water flowed through the condenser, driven by a pump. The cooling water temperature was influenced by the wet-bulb temperature of the ambient air. 

### 2.4. Measurement Instruments and Uncertainty Analysis

During the experimental process, parameters measured included heat source temperature at the evaporator inlet and outlet, heat source temperature at the preheater outlet, heat source volume flow rate, evaporation temperature, pressure and temperature at the turbine inlet and outlet, electric power output of the generator, and cooling water temperature at the condenser inlet and outlet. The sensor layout is shown in Figure 1.

A brief uncertainty analysis was conducted for the primary and calculated parameters listed in Table 4. For all measured variables, the uncertainties were obtained from the specifications of instruments. For the calculated parameters, the uncertainties were estimated using the error propagation method proposed by Moffat [39]. Basically, *R* is the indirectly calculated parameter that can be calculated from several independent and directly measured parameters *X_i_*, as represented by:

(1) $$R=f(X_{1},X_{2},\cdots ,X_{N}).$$

The propagated error $\delta_{R}$ determined by the measurement accuracy of each independent primary parameter $\delta_{X_{i}}$ can be expressed by the root-sum-square method:

(2) $$\delta_{R}=\sqrt{\sum_{1}^{N}{\left(\frac{\partial R}{\partial X_{i}}\right)}^{2}{\left(\delta_{X_{i}}\right)}^{2}}.$$

## 3. Thermodynamic Analysis

**Figure 4:**
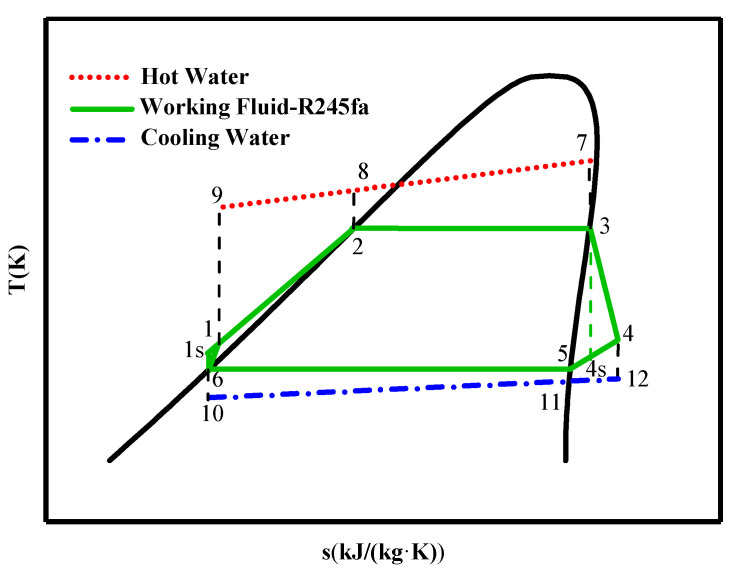
T–s diagram of ORC.

Figure 4 depicts the T–s diagram of the ORC prototype. State parameters of R245fa and water were calculated by REFPROP v9.0 software from the National Institute of Standards and Technology (NIST). 

The high-pressure R245fa liquid was heated in preheater (process 1–2) and evaporator (process 2–3). Heat transfer rates can be calculated by:

(3) $${\dot{Q}}_{preh}={\dot{m}}_{hw}\left(h_{8}-h_{9}\right)$$

(4) $${\dot{Q}}_{evap}={\dot{m}}_{hw}\left(h_{7}-h_{8}\right)$$

where ${\dot{m}}_{hw}$ is the mass flow rate of the heat source; $h_{7}$, $h_{8}$, and $h_{9}$ represent the enthalpy of the heat source at the evaporator inlet and outlet, and at the preheater outlet, respectively.

The high-pressure and high-temperature R245fa vapor passed through the turbine (process 3–4) and converted enthalpy into power. The turbine shaft power can be expressed as:

(5) $${\dot{W}}_{turb}={\dot{m}}_{wf}\left(h_{3}-h_{4}\right)$$

where ${\dot{m}}_{wf}$ is the mass flow rate of R245fa; $h_{3}$ and $h_{4}$ represent the enthalpy of R245fa at the turbine inlet and outlet, respectively, determined by the measured temperature and pressure of R245fa.

The turbine isentropic efficiency can be defined as the ratio of actual power output to power output in the isentropic expansion process, given by:

(6) $$\eta_{is,turb}=\frac{h_{3}-h_{4}}{h_{3}-h_{4s}}$$

where $h_{4s}$ is the ideal enthalpy of R245fa at the turbine outlet in the isentropic expansion process (process 3–4s).

The low-pressure R245fa vapor dissipated heat to the cooling water in the condenser and was condensed into liquid (process 4–6). The heat transfer rate can be specified as:

(7) $${\dot{Q}}_{cond}={\dot{m}}_{cw}\left(h_{12}-h_{10}\right)={\dot{m}}_{cw}c_{p}(T_{12}-T_{10})$$

where ${\dot{m}}_{cw}$ is the mass flow rate of cooling water; $h_{10}$ and $h_{12}$ represent the enthalpy of cooling water at the condenser inlet and outlet, respectively; $c_{p}$ is the specific heat at constant condensing temperature; $T_{10}$ and $T_{12}$ represent the temperature of cooling water at the condenser inlet and outlet, respectively.

The low-pressure and low-temperature R245fa liquid flowed into the preheater driven by the pump (process 6–1). The power consumed by the pump can be calculated by

(8) $${\dot{W}}_{pump}={\dot{m}}_{wf}\left(h_{1}-h_{6}\right)$$

where $h_{6}$ and $h_{1}$ represent the enthalpy of R245fa at the pump inlet and outlet, respectively.

The gross generating efficiency of the ORC system can be defined as:

(9) $$\eta_{gros}=\frac{{\dot{W}}_{elec}}{{\dot{Q}}_{preh}+{\dot{Q}}_{evap}}$$

where ${\dot{W}}_{elec}$ is the electric power output of the generator, which can be directly measured.

The electromechanical efficiency of the generator unit is defined as the ratio of measured electric power output of the generator to turbine shaft power:

(10) $$\eta_{elec\text{-}mech}=\frac{{\dot{W}}_{elec}}{{\dot{W}}_{turb}}.$$

As mentioned in [40], the overall efficiency of the integrated turbine and generator unit can be defined as:

(11) $$\eta_{over\_tg}=\frac{{\dot{W}}_{elec}}{{\dot{m}}_{wf}\left(h_{3}-h_{4s}\right)}.$$

The Carnot cycle provides a theoretical thermodynamic limit for all heat engines and can be expressed as a function of the heat source and cooling source temperatures in Kelvin [13,29,32]:

(12) $$\eta_{carn}=1-\frac{T_{10}}{T_{7}}$$

where $T_{7}$ and $T_{10}$ are the inlet temperatures of heat source and cooling source, respectively.

## 4. Experimental Results and Discussion

In the experiment, at first, the impact of the heat source temperature on ORC performance was evaluated. The heat source temperature increased from 101 to 121 °C, and the cooling water temperature was approximately kept at a constant value of 27 °C. The volume flow rates of heat source and cooling water were set to 105 m^3^/h and 240 m^3^/h, respectively. Subsequently, variations of the ORC performance with heat source volume flow rate were investigated. The heat source volume flow rate varied from 75 to 115 m^3^/h, while the heat source temperature was almost maintained at 116 °C. The cooling water temperature and volume flow rate remained the same as those in the first step.

### 4.1. Effect of Heat Source Temperature on System Performance

**Figure 5:**
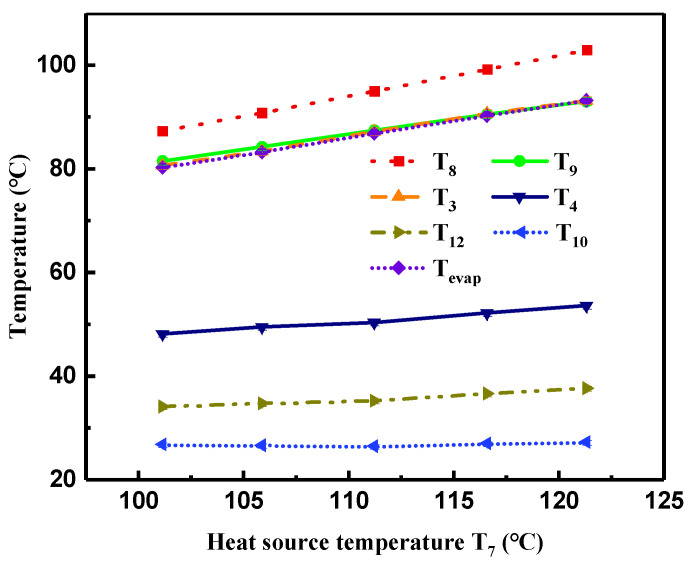
Variations of measured temperature and evaporation temperature with heat source temperature.

Figure 5 illustrates variations of the temperature measured and the evaporation temperature calculated by evaporation pressure with the heat source temperature ($T_{7}$). This Figure indicates that the heat source temperatures at the outlet of the evaporator ($T_{8}$) and preheater ($T_{9}$) as well as the temperature of R245fa at the turbine inlet ($T_{3}$) increased linearly as $T_{7}$ increased. As described in Figure 5, the increment of $T_{7}$ also enlarged the temperature of R245fa at the turbine outlet ($T_{4}$). Although the heat transfer rate in the condenser increased with $T_{7}$, due to the large volume flow rate (${\dot{V}}_{cw}$) of cooling water, the temperature difference ($T_{12}-T_{10}$) of cooling water at the condenser inlet and outlet exhibited a small change, ranging from 7.3 to 10.4 °C. Moreover, $T_{10}$ was approximately constant; therefore, $T_{12}$ presented a slight increase. 

In addition, the range of the temperature difference between the measured $T_{3}$ and the calculated evaporation temperature ($T_{evap}$) varied from −0.05 to 0.37 °C, which might be attributed to measuring error and indicates that the R245fa vapor at the turbine inlet was in a saturated state.

**Figure 6:**
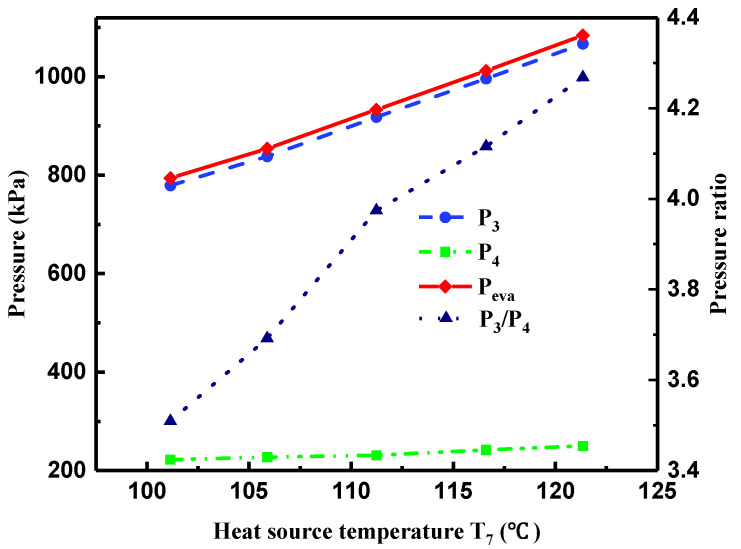
Variations of pressure at the turbine inlet and outlet, pressure ratio, and evaporation pressure with heat source temperature.

Figure 6 presents variations of the pressure of R245fa at the turbine inlet ($P_{3}$) and outlet ($P_{4}$), pressure ratio of $P_{3}$ and $P_{4}$, and evaporation pressure ($P_{evap}$) with $T_{7}$. Owing to the increment of ($T_{7}-T_{8}$) and the constant heat source volume flow rate (${\dot{V}}_{hw}$), the heat transfer rate in the evaporator increased with the increase in $T_{7}$, causing a pronounced increase in $P_{evap}$. Accordingly, $T_{evap}$ presented an increasing trend with the increment of $P_{evap}$, as shown in Figure 5. As $P_{evap}$ rose from 794 to 1084 kPa, $T_{evap}$ increased from 80.24 to 93.2 °C. With the slight increase in ($T_{12}-T_{10}$) and the constant ${\dot{V}}_{cw}$, the gently increasing of the heat transfer rate in the condenser resulted in a mild increase in the condensation pressure. Therefore, the pressure of R245fa at the turbine outlet ($P_{4}$) exhibited a smaller increasing tendency. Consequently, the pressure ratio of $P_{3}$ and $P_{4}$ presented a noticeable growth with $T_{7}$.

**Figure 7:**
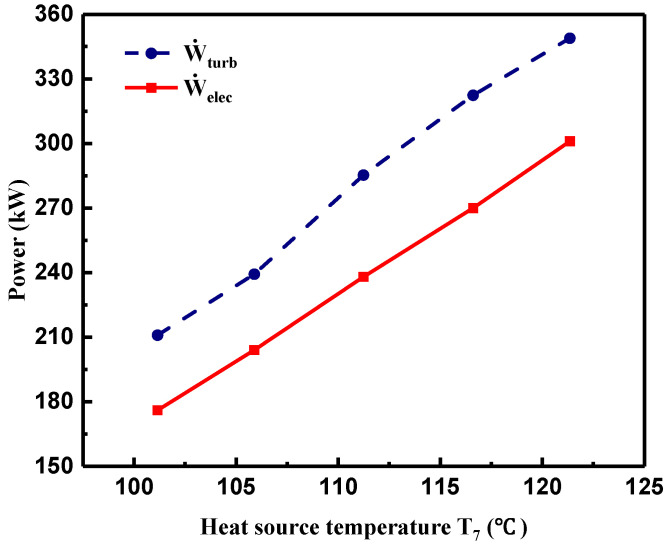
Variations of turbine shaft power and electric power output of generator with heat source temperature.

According to the measured temperature and pressure at the turbine inlet and outlet, the turbine shaft power output (${\dot{W}}_{turb}$) was calculated by Equation (5). As shown in Figure 7, ${\dot{W}}_{turb}$ presented a sharp increment trend with the increasing $T_{7}$ owing to the increment of pressure ratio. Thus, the measured electric power output (${\dot{W}}_{elec}$) increased with the increasing ${\dot{W}}_{turb}$. Due to the energy loss in the power-to-electricity conversion, ${\dot{W}}_{elec}$ was a little lower than ${\dot{W}}_{turb}$. As $T_{7}$ increased from 101 to 121 °C, ${\dot{W}}_{turb}$ increased from 210.9 to 348.9 kW, while ${\dot{W}}_{elec}$ showed a linear increasing trend ranging from 176 to 301 kW, implying that larger electric power output could be achieved with higher heat source temperature.

**Figure 8:**
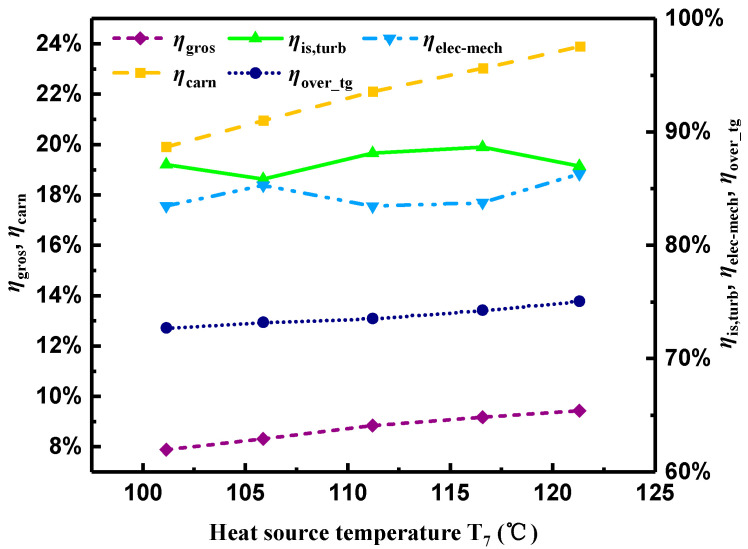
Variations of diverse efficiencies with heat source temperature.

Figure 8 demonstrates variations of the gross generating efficiency ($\eta_{gros}$), turbine isentropic efficiency ($\eta_{is,turb}$), electromechanical efficiency of the generator ($\eta_{elec\text{-}mech}$), overall efficiency ($\eta_{over\_tg}$) of the integrated turbine and generator, and Carnot efficiency ($\eta_{carn}$) with $T_{7}$. Based on the previous discussion, although both the electric power output and total heat transfer rate in the preheater and evaporator were enlarged with the increment of $T_{7}$, the growth rate of the former was higher than that of the latter. As a result, the calculated $\eta_{gros}$ increased, and reached a maximum of 9.4%. $\eta_{carn}$ increased from 19.9 to 23.9% with $T_{7}$. Comparing $\eta_{gros}$ with $\eta_{carn}$, it can be found that $\eta_{carn}$ presented a continuous increasing trend; however, $\eta_{gros}$ showed a slow increasing tendency, and accounted for about 40% of $\eta_{carn}$.

Furthermore, the highest electric power output and gross generating efficiency were reached simultaneously at the highest $T_{7}$. As illustrated in Figure 8, $\eta_{over\_tg}$ presented a slight variation. According to Equation (11), $\eta_{over\_tg}$ is the product of $\eta_{is,turb}$ and $\eta_{elec\text{-}mech}$ in form. $\eta_{over\_tg}$ showed slight growth ranging from 72.7 to 75%. Thus, the contrary changing trend of $\eta_{is,turb}$ and $\eta_{elec\text{-}mech}$ can be explained clearly. To be specific, $\eta_{is,turb}$ and $\eta_{elec\text{-}mech}$ presented a gentle variation from 85.8 to 88.6% and from 83.4 to 86.3%, respectively, with the increment of $T_{7}$. Theoretically, $\eta_{is,turb}$ should have kept increasing when $T_{7}$ increased from 101 to 116.6 °C. However, when $T_{7}$ was at 106 °C, $\eta_{is,turb}$ had a local minimum, which can be seen from Figure 8. This phenomenon was mainly caused by measuring error and error propagation, because the uncertainty of $\eta_{is,turb}$ was a little higher, which was ±6.4%. Furthermore, $\eta_{is,turb}$ reached a peak value of 88.6% when $T_{7}$ was at 116.6 °C. Obviously, the calculated $\eta_{is,turb}$ in the present experiment was higher than those listed in Table 4.

### 4.2. Effect of Heat Source Volume Flow Rate on System Performance

**Figure 9:**
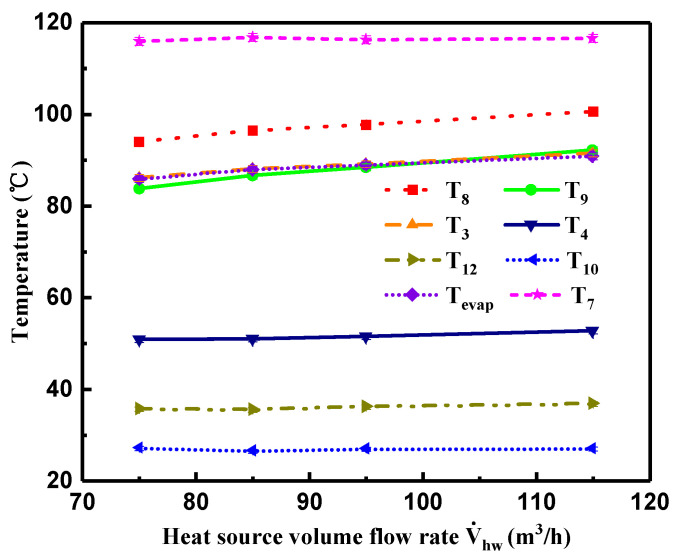
Variations of measured temperatures and evaporation temperature with heat source volume flow rate.

Variations of measured temperature and $T_{evap}$ with heat source volume flow rate (${\dot{V}}_{hw}$) are described in Figure 9. From the Figure, it can be seen that when $T_{7}$, $T_{10}$, and ${\dot{V}}_{cw}$ were almost constant, $T_{8}$, $T_{9}$, and $T_{3}$ showed a gradual increase with higher ${\dot{V}}_{hw}$, whereas $T_{4}$ and $T_{12}$ presented a slight fluctuation with the increment of ${\dot{V}}_{hw}$. With respect to $T_{evap}$, it presented an increasing trend owing to the higher $P_{evap}$. Moreover, the changing tendency of $T_{evap}$ basically coincided with that of $T_{3}$, which suggests that the R245fa vapor was saturated at the turbine inlet.

**Figure 10:**
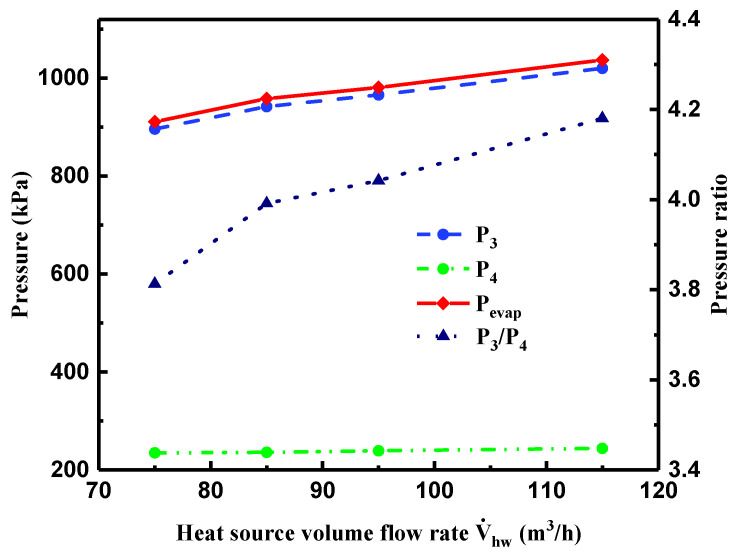
Variations of pressure and pressure ratio at the turbine inlet and outlet, evaporation pressure with heat source volume flow rate.

Figure 10 shows variations of $P_{3}$, $P_{4}$, $P_{evap}$, and the pressure ratio of $P_{3}$ and $P_{4}$ with ${\dot{V}}_{hw}$. When ${\dot{V}}_{hw}$ increased from 75 to 85 m^3^/h, the heat transfer rate in the evaporator was enlarged significantly. However, the heat transfer rate in the evaporator had a slower increase, with ${\dot{V}}_{hw}$ rising from 85 to 115 m^3^/h. As a consequence, $P_{evap}$ presented a trend of noticeable increase at first and then a mild increment, as did $P_{3}$. Furthermore, $P_{evap}$ was slightly higher than $P_{3}$, which can be attributed to the friction loss in pipelines between the evaporator and the turbine during the experimental process. However, $P_{4}$ exhibited a slight variation with the increment of ${\dot{V}}_{hw}$, for the following reasons. As can be seen in Figure 9, on account of the approximately constant value of $T_{10}$, $T_{12}$, and ${\dot{V}}_{hw}$, the heat transfer rate in the condenser had a smaller fluctuation and brought out a flat variation in condensation pressure. Therefore, $P_{4}$ presented a slight variation directly affected by condensation temperature, as shown in Figure 10. Under the comprehensive effects of $P_{3}$ and $P_{4}$, the changing trend of the pressure ratio was derived.

**Figure 11:**
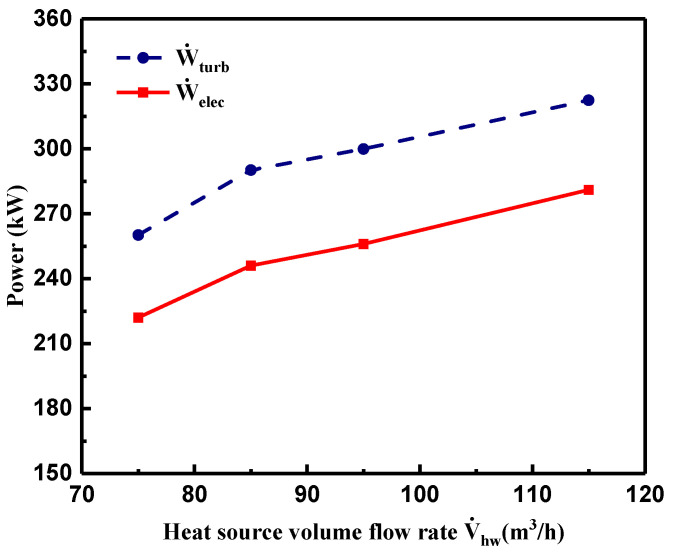
Variations of turbine shaft power and electric power output of generator with heat source volume flow rate.

Figure 11 displays variations of ${\dot{W}}_{turb}$ and ${\dot{W}}_{elec}$ with ${\dot{V}}_{hw}$. Apparently, under the comprehensive effects of the mass flow rate of R245fa, $T_{3}$ and the pressure ratio of $P_{3}$ and $P_{4}$, and ${\dot{W}}_{turb}$ and ${\dot{W}}_{elec}$ were distinctly enhanced with a similar increasing trend, when ${\dot{V}}_{hw}$ increased from 75 to 85 m^3^/h; then ${\dot{W}}_{turb}$ and ${\dot{W}}_{elec}$ had a relatively slower increasing tendency when ${\dot{V}}_{hw}$ varied from 85 to 115 m^3^/h. The maximum turbine shaft power output and electric power output were 322.5 and 281 kW, respectively.

**Figure 12:**
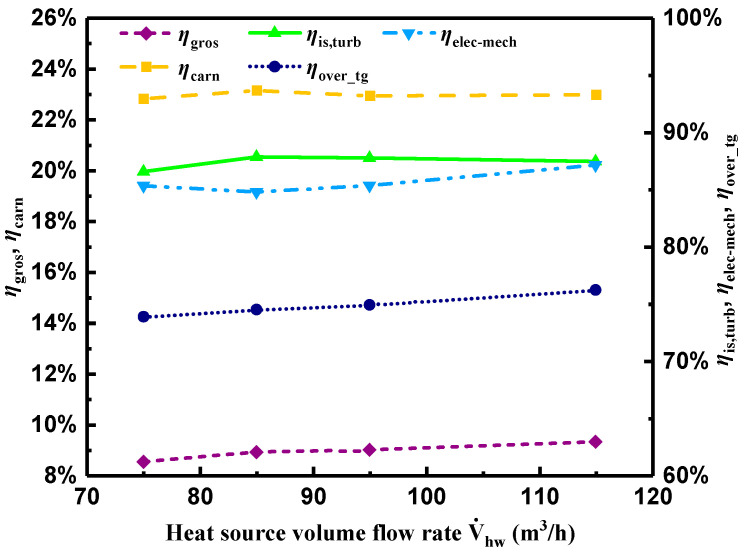
Variations of diverse efficiencies with heat source volume flow rate.

Figure 12 depicts variations of $\eta_{gros}$, $\eta_{is,turb}$, $\eta_{elec\text{-}mech}$, $\eta_{over\_tg}$, and $\eta_{carn}$ with ${\dot{V}}_{hw}$. As can be seen in the Figure, $\eta_{gros}$ presented a gradual increasing trend under the comprehensive effect of electric power output and heat transfer rates in the evaporator and preheater, ranging from 8.5 to 9.3%. However, $\eta_{carn}$ fluctuated from 22.8 to 23% caused by the fluctuation of $T_{7}$ and $T_{10}$. Furthermore, $\eta_{gros}$ was around 40.5% of $\eta_{carn}$, which was higher than the average value obtained from most experimental research by statistics [18].

With regard to $\eta_{over\_tg}$, it showed a gentle increasing tendency ranging from 73.9 to 76.2%. According to Equation (11), $\eta_{is,turb}$ and $\eta_{elec\text{-}mech}$ were in reverse proportion, both presenting a slight fluctuation. The highest isentropic efficiency of 87.9% was achieved when ${\dot{V}}_{hw}$ was 95 m^3^/h and the largest $\eta_{elec\text{-}mech}$ of 87.1% was obtained with the maximum ${\dot{V}}_{hw}$ of 115 m^3^/h. $\eta_{is,turb}$ was higher than those listed in Table 4.

## 5. Conclusions

In order to recover low-grade waste heat in industrial processes, the experimental research on a 300 kW ORC unit with a radial turbine integrated with a three-phase asynchronous generator was conducted. R245fa was employed as working fluid. The influence of heat source temperature, ranging from 101 to 121 °C, and volume flow rate, varying from 75 to 115 m^3^/h, on system performance was investigated. Based on the above discussion, the following conclusions were derived:

- As the heat transfer rates in the evaporator and preheater increased with the increasing heat source temperature or volume flow rate, the heat source temperature at the evaporator and preheater outlet, the temperature of R245fa at the turbine inlet and outlet, and the evaporation temperature of R245fa increased to some extent. However, the cooling water temperature at the condenser outlet showed a relatively slight variation due to the approximately constant operating condition of the cooling source.
- The evaporation pressure and the pressure of R245fa at the turbine inlet exhibited a noticeable increment with higher heat transfer rate in the evaporator, while the pressure of R245fa at the turbine outlet presented a gradual increasing tendency, resulting in the increased electric power output and gross generating efficiency. The highest electric power output and gross generating efficiency were 301 kW and 9.4%, respectively. Higher electric power output yielded higher gross generating efficiency.
- The maximum Carnot efficiency, the theoretical thermodynamic limit of ORC, was 23.9%, which indicates that it is a technology with intrinsic low efficiency. The gross generating efficiency of the ORC in the current experiment accounted for about 40.5% of the Carnot efficiency, which was higher than the average value obtained by statistics. The turbine isentropic efficiency was above 85%. As for improving the system efficiency, regenerative ORC or regenerative extraction ORC could be employed. Furthermore, an economic evaluation would be indispensable when improving the ORC performance.
- Both the turbine isentropic efficiency and electromechanical efficiency of the generator had slight variations with diverse heat source temperature and volume flow rate, but the trends were contrary. The maximum isentropic efficiency of 88.6% and electromechanical efficiency of 87.1% were obtained.
- The overall efficiency of the integrated turbine and generator exhibited a gentle variation, which indicated that it was in a stable operating condition in the experiments.

## Figures and Tables

**Table 1:** Organic Rankine cycle (ORC) classification according to the heat source temperature and power capacity range [8].

Classification	Heat Source Temperature (°C)	Classification	Power Capacity (kW)
Low	<150	Micro	<3
Medium	150–250	Mini	3–50
High	>250	Small	50–500
		Medium	500–5000
		Large	>5000

**Table 2:** Experimental results of ORC reported in detail in published literature (sorted by time).

Authors	Heat Source	Temperature	Heat Source Capacity	Working Fluid	Expander Type	Power Output	Cycle Efficiency	Isentropic Efficiency
Nguyen et al. [19]	Hot water	93 °C	60 kW	n-pentane	Radial turbine	1.44 kW	4.3%	49.8%
Yamamoto et al. [2]	Electric heater	50–80 °C	20 kW	R123	Radial turbine	150 W	1.25%	47.8%
Quoilin et al. [20]	Hot air	101–163 °C	–	R123	Scroll expander	1.8 kW	7.4%	68%
Pei et al. [21]	Hot oil	105 °C	18.3 kW	R123	Radial turbine	1 kW	6.8%	65%
Kang [22]	Steam	77–83 °C	700 kW	R245fa	Radial turbine	32.7 kW	5.22%	78.7%
Zheng et al. [23]	Hot water	40–90 °C	36 kW	R245fa	Piston expander	0.35 kW	5%	43.3%
Han et al. [24]	Hot water	140 °C 150 °C	2 MW	R245fa	Radial turbine	201 kW	–	72.4%
Hsu et al. [25]	Hot Water	80–125 °C	1050 kW	R245fa	Screw expander	50 kW	10.5%	72.5%
Minea [26]	Hot water	85–116 °C	700 kW	R245fa	Screw expander	39.9 kW	7.57%	–
Abadi et al. [27]	Hot water	80–120 °C	110 kW	R245fa/R134a	Scroll expander	1.2 kW	6%	65%
Fu et al. [28]	Hot water	119.2 °C	3788 kW	R245fa	Turbine	225 kW	7.94%	63.7%
Galloni et al. [29]	Hot water	75–95 °C	11 kW	R245fa	Scroll expander	1.2 kW	9.28%	84.9%
Miao et al. [30]	Hot oil	140 °C 160 °C	100 kW	R123	Scroll expander	2.35 kW 3.25 kW	6.39% 5.12%	81%
Muhammad et al. [31]	Steam	100–140 °C	17.4 kW	R245fa	Scroll expander	1.02 kW	5.75%	77.74%
Peris et al. [32]	Hot oil	90–150 °C	390 kW	R245fa	Volumetric expander	36.6 kW	9.4%	70%
Yun et al. [33]	Hot water	120 °C	45 kW	R245fa	Scroll expander	3.4 kW	7.5%	61.4%
Pu et al. [34]	Hot water	<100 °C	–	HFE7100 R245fa	Axial turbine	1.03 kW 1.98 kW	4.01% 4.17%	59.7% 62%
Sung et al. [35]	Hot water	140 °C	2200 kW	R245fa	Radial turbine	177 kW	9.6%	68.1%
Feng et al. [36]	Hot oil	110–140 °C	80 kW	R123	Scroll expander	2.01 kW	3.25%	85.17%
Shao et al. [37]	Hot oil	110–140 °C	55 kW	R123	Radial turbine	1.88 kW	5.7%	83.6%
Ziviani et al. [38]	Hot water	85 °C 110 °C	100 kW	R245fa	Scroll expander	3.75 kW	–	58%

**Table 3:** Thermo–physical properties of R245fa and R123.

Working Fluid	Molecular Weight (g/mol)	T_nb_^1^ (K)	T_cr_^2^ (K)	P_cr_^3^ (kPa)	ODP ^4^	GWP ^5^	ASHRAE ^6^ Safety Group
R245fa	134.05	15.14	154.01	3651	0	858	B1
R123	152.93	27.82	183.68	3662	0.012	120	B1

^1^ T_nb_ is normal boiling temperature; ^2^ T_cr_ is critical temperature; ^3^ P_cr_ is critical pressure; ^4^ ODP is ozone depletion potential; ^5^ GWP is global warming potential; ^6^ ASHRAE is American society of heating, refrigerating, and air-conditioning engineers.

**Table 4:** Parameters measured and the uncertainties of main parameters.

Parameter	Instrument	Measurement Range	Uncertainty
Temperature	WZPK2	−200 to 600 °C	±(0.3 + 0.5% |t|) °C
Pressure	dTRANS	0–25 bar	±0.2%
Electric power	Smart energy meter	N/A	±0.5%
${\dot{W}}_{turb}$			±8.27%
P_3_/P_4_			±0.29%
*η*_carn_			±0.95%
*η*_gros_			±6.1%
*η*_i__s,turb_			±6.4%
*η*_elec–mech_			±8.3%
*η*_over_tg_			±10.5%

## Nomenclature

$\dot{Q}$	heat transfer rate, kW
$\dot{W}$	power, kW
T	temperature, °C
$\dot{V}$	volume flow rate, m^3^/h
$\dot{m}$	mass flow rate, kg/s
h	enthalpy, kJ/kg
Greek symbols	
$\eta_{is,turb}$	isentropic efficiency of turbine
$\eta_{gros}$	gross generating efficiency of ORC system
$\eta_{elec\text{-}mech}$	electromechanical efficiency of generator unit
$\eta_{over\_tg}$	overall efficiency of integrated turbine and generator unit
$\eta_{carn}$	Carnot efficiency
Subscripts	
1–12	state points
hw	hot water
wf	working fluid
cw	cooling water
preh	preheater
evap	evaporator
turb	turbine
cond	condenser
pump	pump
